# Response to: Comment on “Cost-Saving Early Diagnosis of Functional Pain in Nonmalignant Pain: A Noninferiority Study of Diagnostic Accuracy”

**DOI:** 10.1155/2016/4657102

**Published:** 2016-11-29

**Authors:** N. Egloff, R. J. Cámara, C. Merz, B. Wegmann, S. Stauber, R. von Känel

**Affiliations:** ^1^Department of General Internal Medicine, Division of Psychosomatic Medicine, Inselspital, Bern University Hospital, 3010 Bern, Switzerland; ^2^Institute of Medical Biostatistics, Epidemiology, and Informatics, University Medical Center of the Johannes Gutenberg University, Mainz, Obere Zahlbacher Straße 69, 55131 Mainz, Germany; ^3^Heart Failure and Transplantation, Department of Cardiology, Inselspital, Bern University Hospital, 3010 Bern, Switzerland; ^4^Department of Psychosomatic Medicine, Clinic Barmelweid, 5017 Barmelweid, Switzerland; ^5^Department of Neurology, Inselspital, Bern University Hospital, 3010 Bern, Switzerland

It is with great interest that we have read the comment by Toda [1], who refers to our publication “Cost-Saving Early Diagnosis of Functional Pain in Nonmalignant Pain: A Noninferiority Study of Diagnostic Accuracy” [2]. Toda proposes abandoning the differentiation between functional pain and neuropathic pain.

We agree with the author in that we believe that both types of pain are related to the nervous system in an equally tangible way and that both types of pain differ from classic nociceptive pain.

Undeniably, there are also fluent transitions between the two categories of pain [3] and syndromes with combinations of these two pain categories [4].

However, we wish to emphasize that the main difference lies in the pathogenetic causality. By definition, neuropathic pain arises through localized damage or diseases of nerves [5]. According to this definition, neuropathic pain corresponds to a bottom-up dysfunction of the “neural hardware.”

In somatoform-functional pain, however, the functionality of the nervous system is altered as a result of higher order information processes that influence central pain perception. These processes have no structural correlate and can often only be depicted by functional imaging techniques [6].

Information processes, such as biographical imprints, conditioning, and stress can all lead to pain sensitization of the central nervous system on the one hand [7] and to sensitization of the periphery via neuroendocrine top-down mechanisms on the other hand [8]. As our study shows, a resulting generalized hyperalgesia can be used as a core criterion to identify, with high diagnostic accuracy, such hyperperceptive functional pain disorders [2].

Interestingly, patients with functional pain and patients with neuropathic pain do not qualitatively describe their pain with the same words (e.g., agonizing versus burning). Lastly, the causal context of concomitant psychological symptoms is also not quite the same: a neuropathic pain may induce dysphoria and depression as secondary reactions. In contrast, the concept of stress-induced hyperalgesia relies on the evidence that concomitant psychological symptoms (depression, anxiety) accompanying functional pain are a consequence of the same triggering circumstances, that is, long-lasting distress [7]. In our opinion, functional and neuropathic pain should be differentiated. The distinction in pathogenesis implies different therapeutic approaches and also different preventive measures for these two types of pain.
